# Big-team science reveals that patterns of inhibitory control variation in teleost fishes differ from those in mammals and birds

**DOI:** 10.64898/2026.09.22.753528

**Published:** 2026-09-25

**Authors:** Laurent Prétôt, Elia Gatto, Tyrone Lucon-Xiccato, Samina Abedi, Christian Agrillo, Pablo Arechavala-López, Maria P. Arteaga-Avendaño, Leonardo Ávila, Alexis Berry, Benjamin C. Bluck, Culum Brown, Redouan Bshary, María J. Cabrera-Álvarez, Gabriella Dennis, Vanessa Guerrini, Yseult Héjja-Brichard, Kyndal B. Irwin, Nicola Jung, Morgan Leeper, Tamra C. Mendelson, Kasturi Mondal, Inês Neves, Cait Newport, Alessandra Pecunioso, Shane Rance, João L. Saraiva, Ronen Segev, Levi Storks, Justin Yeager, Zegni Triki

## Abstract

Understanding the evolution of cognition is fundamental to understanding the origins and nature of our own cognitive abilities, with cross-species comparisons providing a cornerstone of this endeavor. Yet, comparative cognition so far has largely focused on endothermic vertebrates, particularly mammals and birds, while ectotherms—representing a substantial proportion of vertebrate diversity—remain relatively understudied. Moreover, variation in experimental methods makes existing data difficult to compare across species. Big-team science offers a way to overcome these limitations by enabling standardized cognitive testing across broad taxonomic scales. Here, we apply this approach to fishes, using inhibitory control as a proof of concept for standardized comparative testing in 444 individuals across 22 teleost species spanning 19 genera. We demonstrate that standardized cognitive testing across such diversity is feasible, while also revealing substantial heterogeneity in the sample. Performance varied markedly among species, but, strikingly, showed only a weak phylogenetic signal, contrasting with patterns previously reported in mammals and birds. In addition, we detected great within-species variation, including differences across research groups, suggesting that performance may reflect not only species-level evolutionary history but also ontogenetic experience and the capacity to adjust decision rules to local conditions. Together, our findings highlight the importance of both evolutionary and environmental sources of cognitive variation and demonstrate that extending standardized comparative approaches beyond traditionally studied endotherms can reveal a more dynamic and complex picture of cognitive evolution across the vertebrate lineage.

## Full Text Availability

The license terms selected by the author(s) for this preprint version do not permit archiving in PMC. The full text is available from the preprint server.

